# Below-ground-above-ground Plant-microbial Interactions: Focusing on Soybean, Rhizobacteria and Mycorrhizal Fungi

**DOI:** 10.2174/1874285801812010261

**Published:** 2018-07-31

**Authors:** Nicholas O. Igiehon, Olubukola O. Babalola

**Affiliations:** Food Security and Safety Niche, Faculty of Natural and Agricultural Sciences, Private Mail Bag X2046, North-West University, Mmabatho 2735, South Africa

**Keywords:** Drought, Microbial interactions, Rhizosphere, Soybean, Omic studies, Biofertilizers

## Abstract

**Introduction:**

Organisms seldom exist in isolation and are usually involved in interactions with several hosts and these interactions in conjunction with the physicochemical parameters of the soil affect plant growth and development. Researches into below and aboveground microbial community are unveiling a myriad of intriguing interactions within the rhizosphere, and many of the interactions are facilitated by exudates that are secreted by plants roots. These interactions can be harnessed for beneficial use in agriculture to enhance crop productivity especially in semi-arid and arid environments.

**The Rhizosphere:**

The rhizosphere is the region of soil close to plants roots that contain large number of diverse organisms. Examples of microbial candidates that are found in the rhizosphere include the Arbuscular Mycorrhizal Fungi (AMF) and rhizobacteria. These rhizosphere microorganisms use plant root secretions such as mucilage and flavonoids which are able to influence their diversity and function and also enhance their potential to colonize plants root.

**Natural Interactions between Microorganisms and Plant:**

In the natural environments, plants live in interactions with different microorganisms, which thrive belowground in the rhizosphere and aboveground in the phyllosphere. Some of the plant-microbial interactions (which can be in the form of antagonism, amensalism, parasitism and symbiosis) protect the host plants against detrimental microbial and non-microbial invaders and provide nutrients for plants while others negatively affect plants. These interactions can influence below-ground-above-ground plants’ biomass development thereby playing significant role in sustaining plants. Therefore, understanding microbial interactions within the rhizosphere and phyllosphere is urgent towards farming practices that are less dependent on conventional chemical fertilizers, which have known negative impacts on the environments.

**Below Ground Rhizobacteria Interactions Alleviate Drought Stress:**

Drought stress is one of the major factors militating against agricultural productivity globally and is likely to further increase. Belowground rhizobacteria interactions could play important role in alleviating drought stress in plants. These beneficial rhizobacterial colonize the rhizosphere of plants and impart drought tolerance by up regulation or down regulation of drought responsive genes such as ascorbate peroxidase, S-adenosyl-methionine synthetase, and heat shock protein.

**Insights into Below and above the Ground Microbial Interactions *via* Omic Studies:**

Investigating complex microbial community in the environment is a big challenge. Therefore, omic studies of microorganisms that inhabit the rhizosphere are important since this is where most plant-microbial interactions occur. One of the aims of this review is not to give detailed account of all the present omic techniques, but instead to highlight the current omic techniques that can possibly lead to detection of novel genes and their respective proteins within the rhizosphere which may be of significance in enhancing crop plants (such as soybean) productivity especially in semi-arid and arid environments.

**Future Prospects and Conclusions:**

Plant-microbial interactions are not totally understood, and there is, therefore, the need for further studies on these interactions in order to get more insights that may be useful in sustainable agricultural development. With the emergence of omic techniques, it is now possible to effectively monitor transformations in rhizosphere microbial community together with their effects on plant development. This may pave way for scientists to discover new microbial species that will interact effectively with plants. Such microbial species can be used as biofertilizers and/or bio-pesticides to increase crop yield and enhance global food security.

## 1 INTRODUCTION

Organisms seldom exist in isolation and are usually present in associations with several hosts and the interactions could be in different forms namely: virus *versus* virus, bacterium *versus* bacterium, protozoan *versus* protozoan, fungus *versus* fungus, bacterium *versus* fungus, fungus *versus* plant or animal, bacterium *versus* plant or animal, virus *versus* plant or animal, protozoa *versus* plant or animal, bacterium *versus* fungus *versus* plant or animal, as well as other parasitic and symbiotic associations with unique mechanisms that consolidate the associations which can lead to enhanced host plant growth. The recruitment of a foreign species into a new ecosystem is dependent on the type of host and indigenous microbial community. Generally, an ecosystem with lost species diversity has a high tendency to accept new species or invaders and the invaders have to interact with species in the new ecosystem in order to occupy the niche [1, 2].

**Fig. (1):**
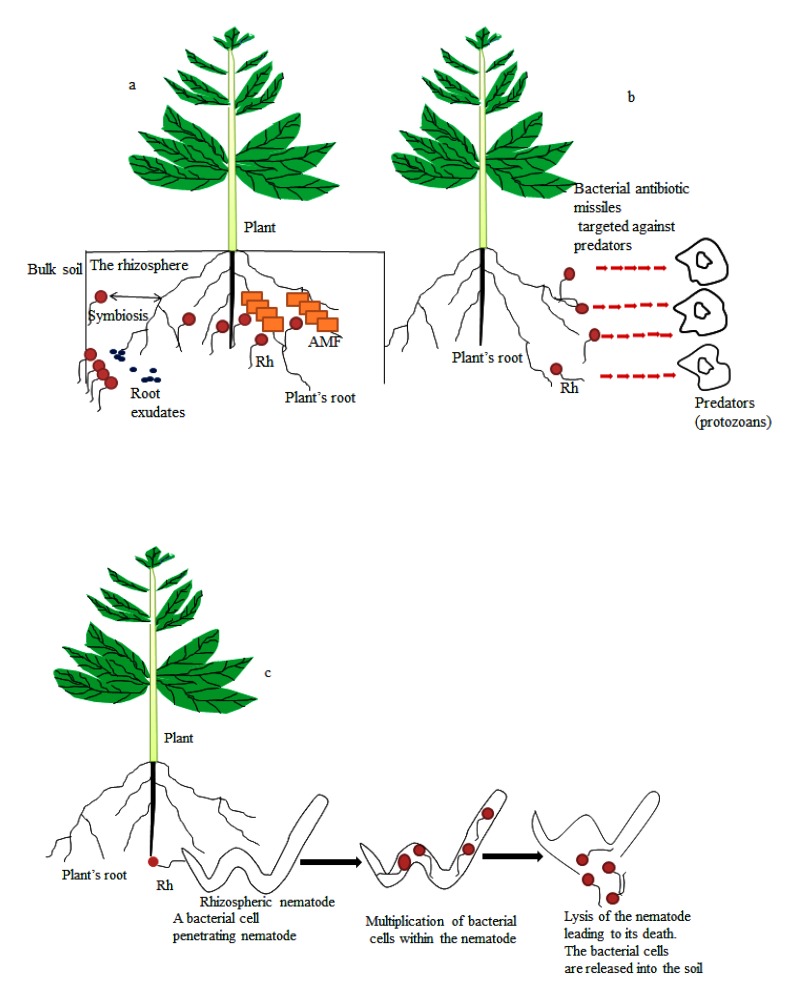
Natural interactions in the rhizosphere. **(a)** symbiotic interactions between plant’s root and Rh/AMF **(b)** amensalistic interaction between antibiotic-producing rhizobacteria and protozoans **(c)** parasitic interaction between the bacterium *Pasteuria penetrans* and root-knots nematode in the rhizosphere. Rh stands for rhizobacteria and AMF stands for arbuscular mycorrhizal fungi.

Research on below and aboveground microbial community are unveiling a myriad of intriguing interactions within the rhizosphere and many of the interactions are facilitated by exudates (Fig. **1**) that are secreted by plant roots [3]. Root exudates play a role in regulating biotic and abiotic functions within the rhizosphere. Some of the functions of the root exudates include: altering soil physicochemical properties, suppressing the proliferation of competing plants and influencing microbial community structure. Particularly amazing are root exudate compounds that are known to mediate symbiotic interactions within the soil. These compounds include monosaccharide (*e.g.* glucose), disaccharide (*e.g.* sucrose), polysaccharide, different types of amino and organic acids such as arginine and benzoic acids. It is also possible for plant roots to secrete “higher-molecular-weight-compounds” such as fatty acids, nucleotides, tannins, alkaloids and vitamins which are known to enhance interactions in the soil [4] particularly those involving rhizobacteria and Arbuscular Mycorrhizal Fungi (AMF).

Rhizobacterial and mycorrhizal fungal interactions have been studied extensively [5, 6]. The main roles of these microorganisms are to provide nutrients to plants, plant growth stimulation, inhibition of phytopathogens growth and soil structure enhancement. In particular, plant symbiotic association involving these bacteria and fungi is a subject of scientific debate. However, interactions involving archaea are not well understood, although they are present in soil rhizosphere where they are involved in bioleaching especially heat loving archaea [7]. Host interactions with viruses are similarly important since viral particles cause several disease conditions in many host and change the bacterial richness and diversity by attacking dominant strains. This means that plant interactions with microorganisms could be positive or negative depending on the species involved.

Considering the enhancement of nutrient status, rhizosphere microorganisms have the capacity to fix nitrogen, obtain iron using siderophores and increase the bioavailability of phosphorus especially by MF [8]. In addition, the introduction of a symbiotic bacterium into the rhizosphere could also impact plant health by enhancing the process of photosynthesis, chlorophyll content as well as assimilation of carbon (IV) oxide [9, 10]. Root endophytes can produce phytohormones in the form of auxins as well as gibberellins which have plant growth promoting traits [11]. There are several host/rhizobacterial interactions which may have beneficial and or disease-causing effects on the host plant. Microbial interactions with plants either in the rhizosphere or plant tissue have also the potential to enhance plant development by releasing phytohormones, cleaning up pollutants and enhancing tolerance to biotic and abiotic stress [12].

Drought stress which may either be short, moderate or very severe, is one of the most detrimental abiotic stresses that have increased in intensity, currently having negative impacts on global food security [13]. By 2050, drought is anticipated to cause severe plant problems for over 50% of the agricultural land [14]. To overcome the effects of drought, some rhizobacteria produce 1-aminocyclopropane-1-carboxylate (ACC) deaminase which breakdown ethylene precursor ACC to 2-oxobutanoate and ammonia thereby reducing ethylene content and eventually minimizing plant stress [15]. Thus, belowground microbial communities can be manipulated towards the selection of microbial assemblages that can tolerate abiotic stress and enhance drought tolerance and health of plants.

This review therefore focuses on recent progresses in microbial interactions with plants. The discussion is largely targeted to AMF and rhizobacteria and their effects on below and aboveground plant community. Similarly, plants’ tolerance to stress by these microbial interactions, insights from omic analyses of the components of these interactions and prospects of harnessing these interactions for agricultural sustainability are highlighted.

## 2 SOIL: THE SITE FOR PLANT MICROBIAL INTERACTIONS

Soil is the region below the earth’s surface composed of both living and nonliving entities for which structure is influenced by both biotic and physicochemical parameters. Biotic factors such as plant nature and plants root enhanced soil structure when bringing soil organic matter (∆ C/N) piercing through the soil matrix (aeration) and the constituent microbial species similarly affect the fertility of the soil, thus may modulate soil pH, structure and other related soil properties. Rhizobacterial existence in the soil might support plant growth by releasing growth enhancing compounds, helping to form firm soil structure, liberating mineral elements from the degradation of organic compounds as well as by forming mutualistic association with plant’s root Fig. (**1a**). Bacteria and MF for instance contribute to the improvement and maintenance of soil structure [16]. It was reported that soil parameters (such as soil pH) are involved in the establishment of microbial community and structure in soil rhizosphere [16, 17] and that changes in microbial community composition could cause transformations in their functions [18] most especially in the rhizosphere.

## 3 THE RHIZOSPHERE: A ZONE OF MICROBIAL INTERACTIONS

The rhizosphere Fig. (**1a**) is the thin zone of soil close to plant roots and harbor a great deal of microorganisms as well as invertebrates [3, 19]. These organisms constantly interact with one another for survival resulting in the establishment of different associations such as commensalism, parasitism, amensalism, saprophytism and symbiosis. Organisms that inhabit the rhizosphere can greatly influence plants’ growth and development [20, 21] thereby playing significant role in sustaining the plants. Some of the microorganisms that are of great benefit to plants in the rhizosphere are rhizobacteria and MF. Taxonomic investigation demonstrated that the rhizosphere species are a subset of bulk soil species [19]. Many studies on microbial communities of the rhizosphere have shown the influence of plants on rhizosphere microbiota. Examples of such studies include soybean [19], *Arabidopsis* [22, 23] and rice [24].

However, it is not totally clear to what degree plants can choose a consistent rhizosphere microbial composition from profoundly varying deposits of bulk soil microbial composition particularly under tropical environments [19]. There are two mechanisms used to explain the choice of plants’ microbial diversity in rhizospheric soil namely neutral and niche based mechanisms. The neutral based mechanism explains that since most species have the same abilities in exploiting niches, microbial composition is influenced by spatial distance between plants due to low recruitment realization and dispersal restriction. The niche based approach shows that environmental changes are related to variation in microbial community composition. Mendes, *et al.* 2014 [19] reported that soybean chooses a particular microbial group in the rhizosphere based on functional characteristics that might be useful to the plant in relation to nutrient uptake and growth enhancement. This selection ability possessed by soybean plant could be explained by niche based hypothesis, showing the strong ability of the plant to determine the richness and abundance of microorganisms in the rhizosphere and other ecological changes that influence microbial diversity.

Species in the rhizosphere mainly migrate from the bulk soil Fig. (**1a**) and environmental changes in the bulk soil could affect the structure and composition of microbial community in the rhizosphere. Rhizosphere microorganisms use nutrients such as mucilage and exudates liberated by plant roots and these nutrients influence the microbial diversity and function within the rhizosphere [12]. Root released flavonoids, different organic acids and cutin monomers that are involved in regulation of plant root-microbial interactions as well as microbial gene expression [25]. It is very possible that many other chemical signals produced and secreted by the root will be recognized and possibly deployed to improve microbial colonization of the root for sustainable agricultural development. It was recently reported that methyl salicylate secreted from the root could trigger root colonization by beneficial *Bacillus subtilis* [26]. Beneficial microorganisms in the rhizosphere vigorously respond to root exudates by tuning their transcriptional machinery toward traits associated with mobility, detoxification, chemotaxis, secondary metabolism, biofilm formation and polysaccharide degradation [27, 28]. Once beneficial microorganisms are established in the rhizosphere, the root exudates might serve as environmental signals to enhance biofilm formation on the surface of the root [29]. Many published articles have excellently described the mechanisms, functions and communication signals involved in root-microbial interactions [4, 30-33].

## 4 NATURAL INTERACTIONS BETWEEN MICROORGANISMS AND PLANTS

Microorganisms interact with plants in the natural environment and such interactions are important in ecosystem functioning. Some of the interactions protect the host plants against pathogens and provide nutrients for the hosts while others are detrimental to the plant. Beneficial microbial interactions in the soil have been found useful in enhancing and improving agricultural crops development Table **1**. Soybean (*Glycine max* L) which is a leguminous plant belonging to the order Fabales, family Leguminosae has the potential of forming symbiotic associations with rhizosphere microbiota [34] including MF. It has been reported that these microbial symbionts support their hosts in the environment.

Therefore, understanding microbial interactions within the rhizosphere is urgent towards farming practices that are less reliant on conventional chemical fertilizers, which have known negative impacts on environmental receptors [34]. Some of the interactions that occur in nature among organisms are highlighted below:

## 4.1 Antagonism

 This is an interaction between two species in an ecosystem in which one species has effect on the other. Some antagonists synthesize enzymes such as cellulases, chitinases, protein and lipid degrading enzymes that have the potential to lyse the cell walls of fungal pathogens [35]. Rhizobacteria are able to reduce the adverse effect of pathogenic microorganisms in the rhizosphere through this interaction. For instance, Firmicutes, Actinobacteria and Proteobacteria play essential role in suppressing the disease caused by root-infecting fungal pathogen *Rhizoctonia solani* and this led to the suggestion that certain interactions are fundamental to disease suppression [36]. A study of antagonistic bacteria to eradicate the effect of *R. solani* on lettuce demonstrated that the bacteria had temporary effects on indigenous rhizobacteria and endophytic fungi [37]. Another report highlighted that the use of antagonistic bacteria on lettuce minimize the disturbance to resident fungal and bacterial species caused when *R. solani* alone was present [38]. Negative effects on symbiotic AMF would explain the unpredictable outcomes of field application of this antagonist [39]. However, it was reported that beneficial bacteria *Pseudomonas fluorescens* F113, which releases antifungal compound 2, 4-diacetylphloroglucinol, was not harmful to AMF *Glomus mosseae* but basically enhanced root colonization by the fungus symbiotic species [40]. Besides bacteria, some fungal species may contribute to mycorrhizal establishment while they inhibit other fungal species. This implies that, there are certain fungi that inhibit other fungal species. Hence, it is essential to establish “fungus specificity of mycorrhizal helper bacteria” since some helper fungal species have been found to enhance mycorrhizal establishment and inhibit other non-helper fungi [41]. Antifungal specificity can therefore be a criterion for a better selection of antagonists as biocontrol agents in the future.

Other rhizobacteria such as *Rhizobium* and *Bacillus* produce siderophores [35] which deprive pathogens of iron acquisition from the environment, thus affecting the existence of the pathogens. This eventually culminates in enhancing plant growth and productivity.

## 4.2 Amensalism

This is an interaction between dissimilar organisms in which one of the organisms leaves detrimental effects on another one. In this type of interaction, chemical released by an organism could damage or kill another organism. Amensalism also known as antibiosis leads to the production of antimicrobial substance by one microorganism which may be detrimental to the other microorganisms. The release of antibiotics is the most commonly used mechanism of amensalism in bacteria against their host pathogens. These antibiotics are “low molecular weight compounds” capable of destroying or hampering the enzymatic and metabolic reactions of pathogenic microorganisms thereby retarding their growth [35]. Since many beneficial microorganisms such as *Pseudomonas* species are confronted with several predators [42]; the production of metabolites detrimental to these predators is crucial. Indeed, the elimination of these bacteria from the soil by these nematodes and other predators will result in loss of diversity of plant growth promoting rhizobacteria and such biodiversity loss may affect plant growth and productivity. However some microorganisms have developed escape strategy by producing secondary metabolites. The bio-production of secondary metabolites might be important in reducing vulnerability of bacteria to bacterivorous (organisms that feed on bacteria) predators Fig. (**1b**). Studies have shown that *Pseudomonas fluorescens* produced 2, 4-diacetylphloroglucinol (2, 4-DAPG) that lyses protists [42]. However, abuse of antibiotic releasing rhizobacteria has the shortcoming of causing the emergence of antibiotic resistant pathogens. In another study, 2, 4-DAPG improved the survival of *P. fluorescens* by enhancing its tolerance to attack by the predatory protozoan *Acanthamoeba castellanii*. On the contrary, Pf-5 and Q8r1-96 strains of *P. fluorescens* deficient in 2, 4-DAPG were resistant to attack by *Naegleria americana* and *Colpoda* species while the gacA-mutants of the bacterial species were susceptible to predation by these predatory protists. These complementary findings indicates that the ability of 2,4-DAPG to serve as escape mechanism from predatory enemy is bacterial strain and/or predator specific [43].

*Bacillus*, *Streptomyces* and *Pseudomonas* produced bioactive lipopeptides able to distort cell membrane integrity resulting in death of microbial pathogens including oomycetes and trophozoites of *N. americana*. Viscosin and massetolide lipopeptide produced by two different strains of *P. fluorescens* protect the bacterial strains from predation by *N. Americana*. Bacterial strains with lipopeptide production potential are described as more tolerant to protozoan enemy than lipopeptide deficient strains. However, both strains have the same growth rate in soil where *N. American* species are absent [43].

## 4.3 Parasitic Interaction

In this interaction, one organism benefits at the expense of the other Table **1**. Metabolomics investigation of the parasitic association between the fungal parasite *Stachybotrys elegans* and *R. solani* revealed the expression of different concentrations of secondary metabolites. In the study, overexpression of parasitic and pyridoxal reductase enzyme gene was observed for *S. elegans* and *R. solani* respectively. In addition, *S. elegans* released enzymes involved in cell wall degradation including trichothecenes and atranones toxins. The trichothecenes produced by *S. elegans* have been reported to have effect on *R. solani* metabolism and development [2, 44]. Trichothecenes are also known to hinder protein synthesis and induce oxidative stress in eukaryotes [2].

The impacts of AMF and bacterial species on nematodes have also been studied. The bacteria *Pasteuria penetrans* was reported to reduce root-knots nematodes growth *via* a parasitic interaction Fig. (**1c**). The bacteria multiply within the infected nematodes resulting in their death and infertility in those that are able to survive. There was also a reduction in the mobility and penetration of young nematodes into plant roots when a large number of bacterial spores glued to the young nematodes in the soil [1].

Plants have also developed their own defense mechanisms to combat pathogens. RNA-seq was recently used to detect plant genes involved in defense responses to the plant root pathogen *Verticillium dahliae* [45]. Additionally, plant-based microarray transcriptomics analyses have unveiled a web of signal transduction pathways activated upon recognition of elicitors with defense signaling compounds and Pathogen-Associated Molecular Patterns (PAMPs) [46] as microorganisms naturally interact with plant roots in the rhizosphere. It would be valuable to encourage the interactions between all organisms in the greenhouse and to search for beneficial rhizosphere microorganisms that may have parasitic and antagonistic impacts on plant pathogens.

## 4.4 Symbiotic Interaction

Symbiotic interaction is a mutualistic association in which the two organisms involved in the association benefit as exemplified in the relationship between leguminous plants Table **1**. and nitrogen fixing bacteria [47]. The conversion of atmospheric nitrogen by nitrogen-fixing bacteria to plant’s utilizable ammonia form is termed nitrogen fixation.

Rhizobacteria do not only assist plant with nitrogen fixation and nutrient acquisition in symbiotic interactions, they also produce different signaling compounds to impact their hosts for improving plant development and tolerance to abiotic and biotic stress. Some rhizobacteria species release different compounds that elicit ISR in plant and these compounds include 2, 3-butanediol (volatile organic chemical), diffusible signal factor diketopiperazines, antibiotics produced by rhizosphere associated pseudomonads, polyketides and lipopeptides produced by rhizosphere associated bacilli, biosurfactants and siderophores [48-50]. The mutualist *B. subtilis* released volatile compounds such as butanediol which was reported to elicit induced systemic resistance by modulating the transcription of sodium ion transporter 1 in plants [48, 51]. Rhizobacteria also release compounds that specifically influence root development by inhibiting elongation of primary root and enhancing lateral root formation. Some of these bacteria including fungi produce auxins which interfere with the signaling of this compound in the root [52]. But, auxins derivatives released by *Piriformospora indica* (root endophyte) do not play role in root enhancement of barley plant; instead, they are needed for biotrophic infection of the plant root [53, 54]. Root zone-associated bacteria and fungi also produce dimethyl disulfide and pyocyanin [55, 56] that regulate plant root development by modulating auxins signaling process.

In addition, AMF interactions with plant roots have been reported to influence the aboveground interaction between plant and herbivore, a process that eventually leads to plants’ protection [57, 58]. AMF establish symbioses with over 80% terrestrial plants Fig. (**1a**) unlike rhizobia which are limited to leguminous plants and *Parasponia* species belonging to the Cannabaceae family [3]. AMF symbioses are mainly important to plants cultivated in phosphorus deficient environment where they aid the plants to access inorganic phosphorus that ordinarily are not bioavailable to plants. Increased productivity of soybean that was symbiotically attached to AMF was attributed to enhanced uptake of phosphorus [59]. AMF can also transport nitrogen and sulfur to host plants, and high level of sulfur content which regulated the “sulfate transporter gene” in a host plant has been previously reviewed [6].

Belowground symbiosis does not only occur between microorganisms and plant, but also there are symbioses that involve interaction between one microorganism and another. The association between beneficial bacteria and AMF is symbiotic since the bacteria aid the mycorrhizal in establishing symbiosis while AMF promote bacterial invasion potential and diversity. However, there are other benefits accrued to such interactions. Symbiotic relationship has also been shown to exist between the bacterium *Burkholderia* and *Rhizopus* (fungus) and it was reported that in the absence of *Burkholderia* species, the fungus *Rhizopus* was unable to sporulate indicating that the fungus rely on compounds produced by the bacterium to survive [2]. This interaction is not well comprehended with respects to the metabolites and processes involved.

## 4.4.1 Below and above Ground Tripartite Symbiotic Impacts: Cost and Benefit

Symbiosis between two different species does not take place in isolation in a natural environment. Symbionts are rather embedded in midst of multiple symbiotic interactions. Multiple interactions are reported to change the health benefit of different plant cultivars involved in the interaction as well as environmental ratio of cost to benefit within a focal interaction. Multiplayer impacts in symbiotic interactions also known as Multiple Symbiotic Impacts (MSIs) are widespread. However, the effects of the impacts differ and depend on several factors. In a symbiotic interaction involving plant host and two microbial partners, these factors include intersection of benefit flowing from the host and microbial symbionts, mutualistic interaction cost, the extent to which the host can sustain the interaction cost and the conserved regions in host genomes that regulate the mutualists [60-62].

**Fig. (2):**
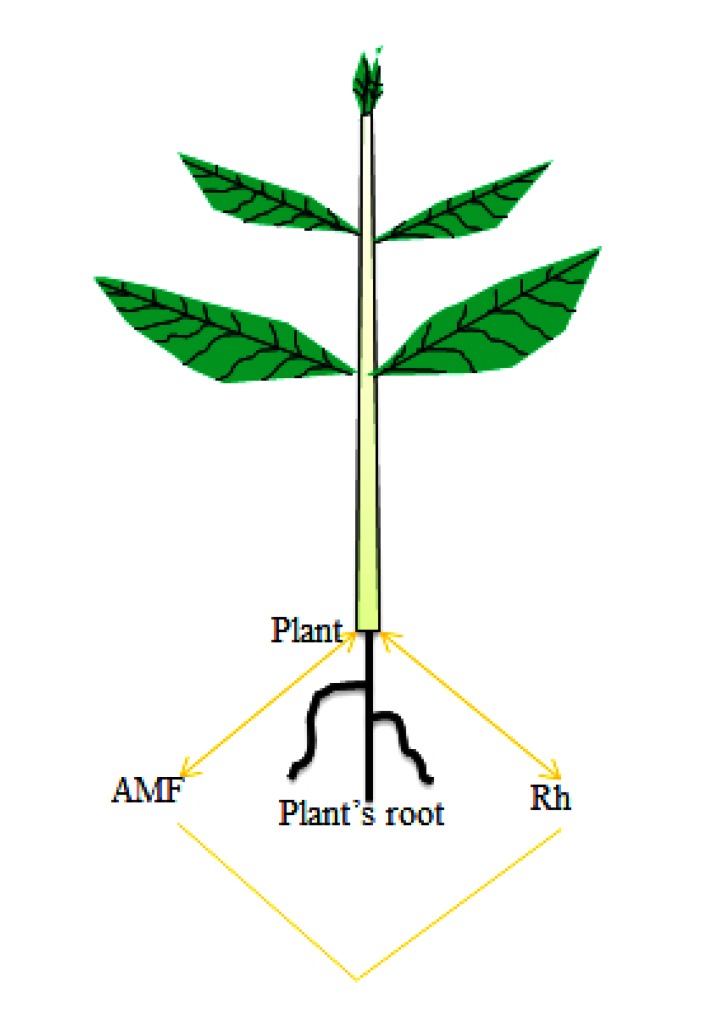
Tripartite interaction involving a leguminous plant (soybean), Rh and AMF. Rh stands for rhizobacteria and AMF stands for arbuscular mycorrhizal fungi.

Studies have shown how several mutualistic microorganisms interacting with the same plant host impact one another. Plants generally undergo symbiotic interactions with several belowground and aboveground symbionts like rhizobacteria, AMF and different endophytic microorganisms. Abd-Alla, El-Enany [63] reported that leguminous plants establish tripartite mutualistic interactions with rhizobacteria species and AMF (Fig. **2**). Plant interactions with several symbionts allow them to obtain enormous benefits which enhance their growth and productivity, resistance to different diseases, pest attacks and abiotic stresses [3, 64-66]. Investigating the mechanisms on how the diversity of these mutualists impact each other including the plant host is not well understood, but such investigation is important to get insights into how hosts are able to simultaneously sustain several mutualistic partners in a MSIs [60].

The cost of sustaining organisms involved in symbiotic relationships could result in tradeoffs among host input [67]. Indeed, it has been observed that in plants that interact with AMF and protective endophytic fungi, the existence of the endophytic fungi triggered a reduction in the level of host colonization by the AMF. If such interactions are important, in an MSI involving plant host in the field, negative correlation will arise between the levels of abundance of the microbial symbionts; that is, increase in the colonization level of one symbiont will lead to a corresponding decrease in the colonization level of the second symbiont. However, if the existence of one of the symbionts enhances nutrient uptake by the plant host and/or increases the amount of carbon substrate release to the second symbiont, then the correlation of the colonization levels between the two symbionts interacting with the same plant will be positive [60]. For instance, ant and coccoid scale are mutualists of *Macaranga* trees; increase in the richness of ant occurs with concomitant increase in the richness of coccoid scales. Perhaps, the high amount of honeydew released by the coccoid scales enhances ant protection [61, 67].

The existence of multispecies interactions involving leguminous plants, rhizobacteria and AMF is critical to understanding the importance of mutualisms in a terrestrial environment Fig. (**2**). Each of the microbial mutualists plays a key role in such interaction by enhancing nutrient uptake, plant yields and community structure [68]. Legumes develop structures in their roots termed nodules which harbor rhizobial species that convert atmospheric nitrogen to the form that can be utilized by the plants. In addition, leguminous crops such as soybean associate with AMF, which have the potential of infecting plant roots and establishing complex intraradical assemblies made up of arbuscules, vesicles, spores and hyphae that assist plants with nutrient uptake. The rhizobacteria and AMF on the other hand utilize the plant’s photosynthetically produced carbon substrates which may determine the extent of root colonization by the microbial symbionts. Co-inoculation of both the rhizobacterial species and AMF results in a synergistic impact on host diversity, development and health, which can be greater than the impact expected from a single inoculation [69]. It is noteworthy that genetic investigations have revealed commonality in plants genome involved in symbiotic relationship and control of AMF and rhizobacteria indicating that such plant-microbial interactions may have evolutionary linkage [70]. Leguminous plants possess unique receptors for discriminating *Rhizobium* and AMF symbionts; even though the symbionts however have common SYM genes that regulate the establishment of symbiotic association.

AMF and rhizobacterial colonization of densely infected plant roots can be controlled in a process called autoregulation [71]. It is evident that AMF influence the persistence and performance of rhizobacterial nodulation and vice versa. AMF have been reported to enhance nodule biomass, reduction in acetylene activity and plant root nodulation richness which may have occurred as a result of increased uptake of phosphorus by the plant [63, 69, 71, 72]. Rhizobacterial inoculation could also affect AMF by either increasing or decreasing their colonization level.

## 5 INFLUENCE OF BELOW GROUND MICROBIOTAS ON ABOVE GROUND INTERACTIONS

**Fig. (3):**
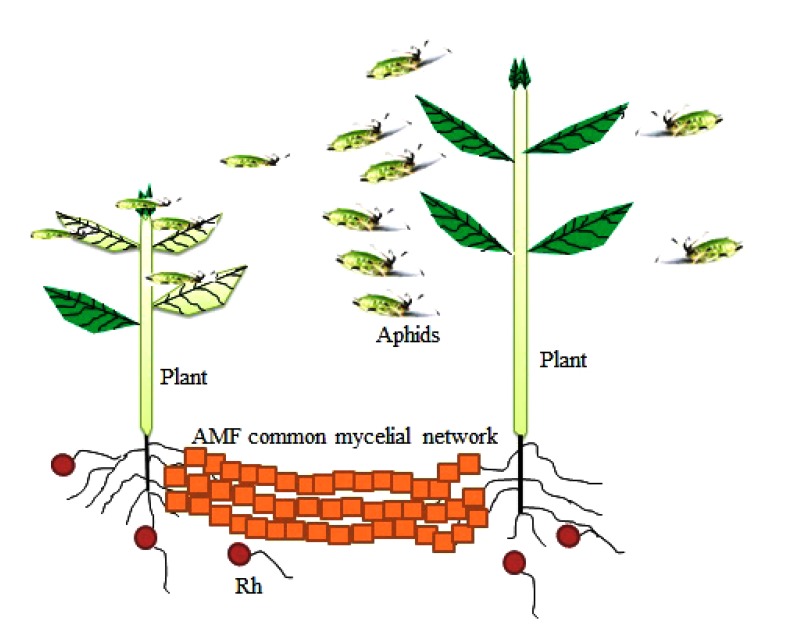
Phyllosphere protection by AMF common mycelial network. Aphids attack on the stunted plant (left) led to the release of modified signaling compound (volatile organic compound) *via* common mycelial system thus preventing their attack on the other plant (right).

There are reports on how interactions in the rhizosphere affect aboveground interactions with pathogens, decomposers, carnivores, mutualists and herbivores [90]. These ‘below-ground-above ground interactions’ in plant can be triggered by changes in water and nutrient uptake or modification in plant defenses [91]. Rhizospheric fungi such as AMF, as well as rhizobacteria (*e.g.* species of *Rhizobium Bacillus* and *Pseudomonas*) can elicit the systemic host immune responses that naturally help the plant to resist to pathogens and pests from the aboveground plant biomass [92]. In particular, the plant response to the aboveground herbivore may be due to the interaction of the plant with belowground AMF (Fig. **3**).

On the other hand, induced defense response in the phyllosphere could spread to the roots and affect rhizosphere microorganisms [93]. Rhizobacteria trigger systemic resistance in plants by activating defense responses further exemplified upon pathogen attack. These immune responses are modulated by plant-derived jasmonic acid, ethylene and salicyclic acid leading to secondary metabolites production, oxidative burst as well as cell wall reinforcement. For instance, the type and concentration of secondary metabolites that are detrimental to herbivores were modulated in the plant by rhizosphere microbiotas [94]. Rhizobacteria do not only facilitate the production of defense metabolites such as glucosinolates, they also enhance plant metabolites production with unknown structures and functions [95].

## 5.1 Belowground AMF Interactions Trigger Phyllosphere Protection

The phyllosphere is the part of the plant that is above the ground level mainly the region around the leaves. Studies have shown how belowground associations can affect aboveground (phyllosphere) interactions with herbivores and carnivores [3]. AMF, *Bacillus* and *Pseudomonas* are able to elicit in plants a systemic resistance response that is detrimental to various disease-causing microorganisms [3, 92]. A study has shown that interplant associations by means of common mycelial systems resulted in high level of resistance to disease, protective enzyme responses and expression of genes that encodes defensive proteins in un-infected or healthy tomato plants (*Lycopersicon esculentum* Mill) linked to diseased plants (*Alternaria solani*) with leaf early blight. This implies that there was possibly transfer of fungal disease protective signals (volatile organic compounds) through the mycelial network to the tomato plant [96]. The systemic resistance (regulated by the host plant hormones) induced by many non-pathogenic rhizobacteria results from elicitation of different defense reactions upon attack by pathogens [3].

If common mycelial networks could facilitate signaling compound’s availability, there is therefore the possibility of AMF facilitating plant defenses to herbivore. Similar effects on these insects’ enemies are also possible since the insects and their enemies react differently to Volatile Organic Compounds (VOCs) released by plants. Herbivores (such as aphids) use VOCs as signals for detecting host plants. However, upon attack, the compositions of subsequent VOCs liberated change which repel other herbivores and attract parasitoid wasps (the herbivore enemies) that destroy the insect [97, 98]. VOCs released by attacked plants are always generated systemically and transferred aerially between plants and transported to the rhizosphere through the roots. Common mycelial networks therefore enhance the transfer of signaling compounds from herbivore attacked plant to un-infested plant resulting in its protection from the insects (Fig. **3**).

Such microbially induced plants protection against herbivore attack guaranties the aboveground biomass and therefore this microbially induced resistance enhances the aboveground plant biomass and could help to minimize the use of expensive and less ecofriendly conventional chemical insecticides. Therefore, from a pragmatic view point, more insights into mycorrhizal functions in plant defense immunity might help to enhance food security [57].

## 6 BELOW GROUND RHIZOBACTERIA INTERACTIONS ALLEVIATE DROUGHT STRESS

Abiotic stresses are some of the factors militating against crop cultivation which may affect their development and yield. The effects of these stresses can be curtailed by belowground microbial interactions since some microorganisms influence the physical and chemical parameters of rhizospheric soil while others may enhance agricultural crops protection to environmental stresses including drought, heavy metal pollutants, salinity and heat . Examples of these microorganisms are bacteria in the rhizobacterial group. The specificity and level of establishment of these plant-microbial interactions are not absolutely explicit and more research is therefore needed to decipher the interactive mechanisms, determinant factors of specificity and how microorganisms confer/amplify plant tolerance to drought environments .

One of the ways to understand the impacts of abiotic stresses on plants is through omic studies which help to elucidate plant microbial processes that can be harnessed for biotechnological purposes. At the transcriptomic level, it was observed that the bacterium *Paenibacillus polymyxa* B2 enhanced the drought tolerance of *A. thaliana* in good correlation with overexpression of some drought responsive genes compared to un-inoculated plants [99]. With the aid of differential display polymerase chain reaction and 2-D polyacrylamide gel electrophoresis, six drought stress proteins (pathogenesis-related protein, adenosine kinase, vacuolar H^+^-ATPase, dehydrin-like protein, S-adenosylmethionine synthetase, early nodulin ENOD18) were expressed in pepper plants amended with *Bacillus licheniformis* under drought stressed condition [76]. Also, quantitative polymerase chain reaction (qPCR) revealed the *Bacillus amyloliquefaciens* 5113 and *Azospirillum brasilense* priming effects on the expression of drought responsive genes [such as ascorbate peroxidase (APX1), S-adenosyl-methionine synthetase(SAMS1) and heat shock protein (HSP17)] and on some enzyme activities involved in enhancing drought stress of wheat leaves [14]. Illumina sequencing (HiSeq 2000 system) revealed drought responsive genes in sugar cane inoculated with the diazotroph *Gluconacetobacter diazotrophicus* PAL5. Enhanced plant acclimatization to arid environment through AMF interaction is connected to the fungal ability to enhance plant nutrient and water uptakes resulting in an increase in the plant tissue water content. Thus, AMF interactions enable plants to acclimatize to drought conditions through different mechanisms which comprise nutritional, morphological, and physiological mechanisms [100]. Although findings have shown how these fungi confer drought tolerance to plant, more information is needed to unveil the whole mechanisms associated with such process.

In addition to single inoculation of rhizobacterial species, a further inoculation with AMF enhances plant drought tolerance. Inoculation of common bean (*Phaseolus vulgaris* L.) with *P. polymyxa*-DSM36, *P. polymyxa*-Loutit strain and *Rhizobium tropici*-CIAT 899 resulted in greater growth compared to single inoculation with *Rhizobium* species. Moreover, combined inoculation increased the number and weight of nodules of the plant under drought stress environment compared to inoculation with *Rhizobium* alone [101]. Under arid and semi-arid conditions, AMF and *Pseudomonas mendocina* significantly improved root phosphatase activity and proline accumulation in lettuce leaves [102]. Subsequent co-inoculation with *Pseudomonas* species and *Azotobacter chrocoocum* reduced drought stress in wheat through increased modifications of the anatomical structures such as mesophyll and phloem tissues, thickness of epidermis and diameter of xylem vessel whereas water deficiency had negative effects on the anatomical tissues of the control [103]. Bacterial consortium of *Bacillus cereus* AR156, *Serratia* species and *B. subtilis* SM21 enhanced drought resistance in cucumber plants. After exposing the plant to drought for 13 days, the plants treated with the bacterial isolates showed darker green leaves with mild symptoms, increased chlorophyll and proline content and decreased relative electrical conductivity. The bacterial consortium treated cucumber plants also enhanced the superoxide dismutase activity and alleviated the drought-activated down-regulation of genes coding for ribulose-1, 5-bisphosphate carboxyl/oxygenase (Rubisco) large and small subunits and ascorbate peroxidase in the leaves of cucumber plants [104]. Microbial consortium containing exopolysaccharide producing bacterial isolates exhibited more potential to drought tolerance in maize compared to single bacterial strain [105]. Rhizobacteria consortia reduced drought stress by minimizing oxidative damage and increasing proline content in rice plants cultivated under drought condition which resulted in enhanced plants growth. These findings show that beneficial plant-microbial interaction under drought stressed condition can be encouraged by the introduction of drought resistant microbial isolates that have the potential to enhance plant metabolic plasticity and to maintain the growth of plants.

## 7 INFLUENCE OF POSITIVE AND NEGATIVE INTERACTIONS ON PLANT AND MICROBIAL DIVERSITY

The associations between plants and rhizosphere microorganisms are dynamic and complicated [3, 106]. Although, belowground interaction can maintain or increase aboveground productivity, compounds generated in the aboveground biomass can have significant positive or negative effects on the belowground microbial diversity and vice versa. In particular, the immune defense structure of plants is believed to play a role in shaping microbial structure. Mutant strain of *A. thaliana* that lacked acquired immunity exhibited microbial community structure different from that of the wild type, but chemical activation of the acquired immune system did not cause noticeable change in the bacterial community in the rhizosphere. Also, reduction in the richness and abundance of endophytes was observed as a result of defense mechanism triggered by salicylic acid in the phyllosphere of *A. thaliana* while plants without jasmonate-triggered defense revealed greater diversity of epiphytes. These results suggest that the distinct plant immune pathways differentially impact soil microbial community and structure especially certain groups of bacteria [3].

The production of plant hormones such as indole-3-acetic acid and gibberellins are common among rhizobacteria species and they are known to enhance plant growth. *Pseudomonas syringae* releases compounds similar to hormones that hinder ethylene and jasmonate signaling thereby allowing pathogen to gain access into the plant through the resultant effect of stomatal opening [3]. On the other hand, many chemical compounds generated by plants enhance specific interactions in certain microbial groups. In particular, flavonoids cause different responses in MF, rhizobacteria, root pathogens and some plants [107] while strigolactones produced in low concentrations from plant roots stimulate MF hyphal branching and parasitic plant (*e.g.* *Orobanche* spp.) seed germination [3, 106]. Glucosinolates generated by transgenic *Arabidopsis* changed the microbial communities in the root and rhizosphere. Alkaloids, terpenoids and phenolics are commonly produced by plants. As an illustration, the order Brassicales and *Arabidopsis* produce glucosinolates, but *Avena strigosa* commonly known as oat produces avenacins which are effective against a wide range of fungi. Mutant oat deficient in avenacins exhibits different rhizosphere culturable fungal species which are more vulnerable to pathogenic fungi than the wild type [106]. Upon analytical investigation, there was amazingly no significant variation in the rhizosphere fungal diversity between the mutant oat and the wild type. However, mutant genotype deficient in avenacins seriously affected eukaryotic community of Amoeba including Alveolata without having any effects on bacteria [106]. This suggests that precise changes in plants metabolism could have serious impacts on plant associated microorganisms.

## 8 INSIGHTS INTO BELOW AND ABOVE GROUND MICROBIAL INTERACTIONS *VIA* OMIC STUDIES

Plants establish interactions with several different microorganisms and a large proportion of these exceedingly complex microbial groups have not been characterized. In particular, it was recently reported that only 7% of all terrestrial fungi have been characterized [108] and a significant fraction of the unidentified fungi interact with plant in different ways. Investigating complex microbial community in the environment is a big challenge. Therefore, omic studies of microorganisms that inhabit the rhizosphere are crucial because this is the region where most plant-microbial interactions occur and this technology could help to discover novel beneficial plant-microbial interactions that may be useful to alleviate drought stress of plants.

Real-time application of metagenomic, metatranscriptomic and transcriptomic to rhizosphere microorganisms may possibly provide more insights into complex microbial interactions [36, 109, 110]. A technical method that merges PhyloChip metagenomics of the rhizosphere microbiota with culture-based transcriptomic analyses unveiled over 33000 archaeal and bacterial species in the rhizosphere of plants cultivated in a disease-suppressive soil [21]. In addition, data from structural genes, functional genes and protein analyses and metabolomic investigations may be combined statistically that will help to detect vital biological functions and make projections *via* modeling. This strategy was recently utilized to gain insights into interaction involving ectomycorrhizal *Laccaria bicolor* and *Populus tremuloides* roots. Transcriptomic profiling of matured ectomycorrhizal *via* next generation sequencing technique resulted in the detection of transcripts which were matched to definite metabolic channels and used to establish the fungal metabolome model. The model was used to project that other metabolites such as glycine and glutamate produced by *L. bicolor* might be utilized by *P. tremuloides* which in return, supplies carbon substrates such as glucose and fructose to the AMF [36].

Furthermore, omic will be useful in detecting the roles of different genes, RNA, proteins and metabolites involved in plant-microbial interactions which would eventually unveil important processes that strengthen these interactions. This approach could simply be accomplished by establishing ‘genome-scale models’ that are appropriate for metabolic flux analyses in which microbial groups might be seen as a single interacting super-organisms while interacting plant’s ‘genomic-scale models’ integrate a certain level of division and differentiate metabolic processes [111, 112]. This type of model has been constructed for *Arabidopsis* and some bacterial spp.

## 9 FUTURE PROSPECTS

According to Babalola [16], interactions between plant and microbial species are not exhaustively understood. Hence, the need for more studies on plant microbial interactions is becoming more pressing as this will give useful insights that may be important in sustainable agriculture development. Also, knowledge of such interactions can be useful in the development of microbial inoculants [113] with growth enhancing potentials. To achieve this feat and discover other plant microbial interactive benefits, several facts have to be understood since considerable numbers of factors are involved in interactions relating to microorganisms, host plant, soil matrix and other environmental components. In addition, the formation of common mycelial network by AMF which have insecticidal potential is an emerging area of study that could be harnessed for the production of bio-insecticides [57, 114]. This will help to minimize the detrimental effects of chemical insecticidal compounds in the environment.

Studies have also depicted that plant-microbial interactions could also play a role in sustainable agriculture by helping to control a wide range of pathogens thereby enhancing crop health, productivity, yield and consequently boosting global food security [115]. However, more knowledge on the physiology of these useful interactive microorganisms, microbial-cultivar acclimatization relationships, and microbial inoculant introduction or delivery techniques is needed. Also required is how inoculated beneficial microbial populations and their activities could be maintained in the soil rhizosphere [11].

Researches into microbial ecological interactions have resulted in significant outcomes biotechnologically [2]. Thus, there is the prospect of developing more useful and beneficial compounds from complex microbial interactions in the ecosystem that could be relevant in agriculture. More knowledge is similarly needed as regards the whole mechanisms involved in interactions between microorganisms and host plants and for this to be accomplished; the establishment of *in vitro* and *in vivo* techniques or models is paramount [36].

## CONCLUSIONS

Plant roots are extremely important for mineral nutrient uptake and productivity, and there is therefore the need to continue study on plant interactions with soil microorganisms. Similarly, more attention should be paid to field application of these microorganisms as biofertilizers and/or bio-pesticides so as to abate the negative effects of conventional chemical fertilizers and pesticides, increase crop yield and enhance global food security. With the emergence of advanced omic technologies, it is now possible to effectively monitor transformations in rhizosphere microbial community together with their effects on plant development. This may pave way for scientists to develop discover microbial species that will interact effectively with plants.

## Figures and Tables

**Table 1:** Benefits of microbial interactions on plants under well-watered and drought stress conditions.

**Biotic Factor**	**Interaction Type/benefit**	**References**
*Pseudomonas putida* and soybean (*G.max*)	Mutualistic interaction between the bacterium *P. putida* and soybean (*G. max*) under drought and saline conditions. Secretion of gibberellins by the bacterium enhanced soybean growth	[73]
*Rhizobium etli* 12a3 and common bean (*Phaseolus vulgaris*)	Mutualistic interaction between *R. etli* 12a3 and common bean (*P. vulgaris*) under drought stress condition. The bacterium enhanced plant tolerant ability of the bean plant by causing increase in proline accumulation	[47]
***Bradyrhizobium diazoefficiens*** and***Aeschynomene*** ***afraspera***	Symbiotic interaction with plant under well-watered condition. The microorganism enhanced host plant growth by fixing atmospheric nitrogen	[2, 74]
*Rhizobium* spp. and soybean (*G. max*)	Symbiotic interaction with plant under well-watered condition. *Rhizobium* inoculation with AMF prevented red crown rot disease of soybean (*G. max*) during symbiotic interaction	[64]
AMF and host plant	Symbiotic relationship with the host. Host plant protection against herbivore attack through common mycelial network in well-watered soil. The AMF’s common mycelial network led to the production of volatile organic compounds that attract parasitoid wasps (herbivore enemies) that destroyed the herbivore	[75]
*Bacillus licheniformis* K11 and pepper plant	Mutualistic interaction between the bacterium *B. licheniformis* and pepper plant under drought stress conditions. Accumulation of drought stress proteins and RNA in the bacterial inoculated plants made them to survive better than the non-inoculated pepper plants	[76]
Rice and species of *Burkholderia* and *Rhizopus*	Symbiotic and pathogenic interaction with plant under well-watered condition. The fungus was unable to produce spores in the absence of the endosymbiont. The endosymbiont produces rhizoxin (the causative agent of seedling blight of rice) and phytotoxin. The fungus stimulates the endosymbiont growth	[77]
*R. solani* and *S. elegans*	Parasitic relationship with the host under well-watered condition. The toxins released by the parasite (*S. elegans*) caused changes in *R. solani* growth and metabolic process leading to down regulation in the biosynthesis of several antimicrobial substances by *R. solani*	[44]
Mycorrhizal fungus *Glomus mosseae* and soybean (*G. max*)	Mutualistic interaction between the fungus *G. mosseae* and soybean (*G. max*) under drought stress condition. The fungus protected the plant against premature nodule senescence induced by drought stress by inducing higher glutathione reductase (GR) activity in soybean root and nodules which may have contributed to decreased oxidative damage to biomolecules involved in early nodules senescence	[78]
*Erysiphe pisi* and *Pisum sativum*	Pathogenic interaction under well-watered condition. The fungus (*E. pisi*) upregulated the gene coding for invertase and decreased the starch content of the host plant (*P. sativum*)	[79]
*Azospirillum* sp. And wheat plant	Mutualistic interaction between *Azospirillum* sp. and wheat plant under drought condition. IAA improved wheat root development and uptake of nutrients and water under drought stress	[80]
*Blumeria graminis and Pisum sativum*	Pathogenic interaction. The fungus (*B. graminis*) caused change in sugar content and down-regulation of photosynthesis in the host plant (*P. sativum*). under well-watered condition ‘The depression in photosynthesis was not only due to cell death and the effective removal of green leaf area, but also to an alteration in host metabolism’	[81]
A consortium of *Mesorhizobium ciceri* (CR-30 and CR-39), *Rhizobium phaseoli* (MR-2), and *Rhizobium leguminosarum* (LR-30) and wheat plant	Mutualistic interaction between the bacterial consortium and wheat plant under drought stress condition. The wheat plant growth, drought tolerance index and biomass were improved by IAA produced by the bacterial consortium	[82]
*Bacillus subtilis* QST713	Amensalistic interaction under well-watered condition. The bacterium produced antibiotic (iturin A) that helps to control damping off disease caused by *Botrytis cinerea* and *R. solani* in host plants	[83]
A Consortium of AMF and *Bacillus thuringiensis*/*Pseudomonas* and *Trifolium repens*	Mutualistic interaction between the microbial species (fungi and bacteria) and the plant *T. repens* in a natural arid soil under drought conditions. AMF/bacterial inoculation significantly enhanced the plant growth by increasing plant’s nutrient uptake and relative water content	[84]
*Pantoea* *agglomerans* C9-1	Amensalistic interaction under well-watered condition. *P. agglomerans* produced herbicolin (antimicrobial substance) effective *against Erwinia amylovora*, the causative agent of fire blight in plants	[85]
A consortium of exotic *Glomus intraradices*, *G. deserticola* and *G. mosseae* and the plant *Juniperus oxycederus*	Mutualisic interaction between the exotic mycorrhizal fungal consortium and the plant *Juniperus oxycederus* under drought stress conditions. Mycorrhizal fungal consortium increased root and shoot nitrate reductase by 38 and 188% respectively with respect to the plant neither treated with composted sewage sludge (SS). ‘Both the plants inoculated with exotic AM fungi and the plants grown with composted SS developed additional mechanisms to avoid oxidative damage produced under water-shortage conditions’	[86]
*Pseudomonas aeruginosa* Migula 7NSK2 and tomato plants	Mutualistic/antagonistic interaction. The bacterium produced pyochelin siderophore that protect tomato plants from the parasitic oomycetes (*Pythium* spp.) under well-watered condition	[87]
*Heteroconium chaetospira* and Chinese cabbage	Mutualistic interaction between the fungus *H. chaetospira* and the host plant Chinese cabbage under well-watered condition. *H. chaetospira* was found to suppress club-root disease (caused by soil-borne protozoan *Plasmodiophora brassicae*) on Chinese cabbage and enhanced the aboveground plant biomass of the plant in non sterile soil. The hyphae of the fungus colonized the root tissues of the plant without causing any external effects on the plant	[88]
*Azospirillum lipoferum* and maize	Mutualistic interaction between the bacterium *Azospirillum lipoferum* and maize under drought condition. Gibberellins produced by the bacterium increased abscisic acid levels and alleviated drought stress	[89]

## LIST OF ABBREVIATIONS

AMF: = Arbuscular Mycorrhizal Fungi
MF: = Mycorrhizal Fungi
ACC: = 1-aminocyclopropane-1-carboxylate
2, 4-DAPG: = 2, 4-diacetylphloroglucinol
RNA: = Ribonucleic Acid
PAMPs: = Pathogen-Associated Molecular Patterns
ISR: = Induced Systemic Resistance
MSIs: = Multiple Symbiotic Impacts
IAA: = Indole-3-Acetic Acid
SS: = Sewage Sludge
VOCs: = Volatile Organic Compounds
Rh: = Rhizobacteria
qPCR: = Quantitative Polymerase Chain Reaction
APX1: = Ascorbate Peroxidase
SAMS1: = S-adenosyl-methionine synthetase
HSP17.8: = Heat Shock Protein
spp.: = Species
